# Comprehensive Behavioral Analysis of Activating Transcription Factor 5-Deficient Mice

**DOI:** 10.3389/fnbeh.2017.00125

**Published:** 2017-07-11

**Authors:** Mariko Umemura, Tae Ogura, Ayako Matsuzaki, Haruo Nakano, Keizo Takao, Tsuyoshi Miyakawa, Yuji Takahashi

**Affiliations:** ^1^Laboratory of Environmental Molecular Physiology, School of Life Sciences, Tokyo University of Pharmacy and Life Sciences Hachioji, Japan; ^2^Section of Behavior Patterns, Center for Genetic Analysis of Behavior, National Institute for Physiological Sciences Okazaki, Japan; ^3^Life Science Research Center, University of Toyama Toyama, Japan; ^4^Division of Systems Medical Science, Institute for Comprehensive Medical Science, Fujita Health University Toyoake, Japan

**Keywords:** ATF5, transcription factor, knockout mouse, behavior, hyperactivity, anxiety-like behavior, psychiatric disorders

## Abstract

Activating transcription factor 5 (ATF5) is a member of the CREB/ATF family of basic leucine zipper transcription factors. We previously reported that ATF5-deficient (ATF5^-/-^) mice demonstrated abnormal olfactory bulb development due to impaired interneuron supply. Furthermore, ATF5^-/-^ mice were less aggressive than ATF5^+/+^ mice. Although ATF5 is widely expressed in the brain, and involved in the regulation of proliferation and development of neurons, the physiological role of ATF5 in the higher brain remains unknown. Our objective was to investigate the physiological role of ATF5 in the higher brain. We performed a comprehensive behavioral analysis using ATF5^-/-^ mice and wild type littermates. ATF5^-/-^ mice exhibited abnormal locomotor activity in the open field test. They also exhibited abnormal anxiety-like behavior in the light/dark transition test and open field test. Furthermore, ATF5^-/-^ mice displayed reduced social interaction in the Crawley’s social interaction test and increased pain sensitivity in the hot plate test compared with wild type. Finally, behavioral flexibility was reduced in the T-maze test in ATF5^-/-^ mice compared with wild type. In addition, we demonstrated that ATF5^-/-^ mice display disturbances of monoamine neurotransmitter levels in several brain regions. These results indicate that ATF5 deficiency elicits abnormal behaviors and the disturbance of monoamine neurotransmitter levels in the brain. The behavioral abnormalities of ATF5^-/-^ mice may be due to the disturbance of monoamine levels. Taken together, these findings suggest that ATF5^-/-^ mice may be a unique animal model of some psychiatric disorders.

## Introduction

Activating transcription factor 5 (ATF5) is a member of the cAMP response element binding protein (CREB)/ATF family of basic leucine zipper transcription factors. ATF5 is widely expressed in the brain, liver, main olfactory epithelium (MOE), and vomeronasal epithelium (VNE) (Persengiev et al., 2002; Angelastro et al., 2003; Wang et al., 2012; Nakano et al., 2016). It has been reported that ATF5 regulates cell differentiation, survival, and apoptosis (Persengiev et al., 2002; Angelastro et al., 2003, 2005; Greene et al., 2009; Dluzen et al., 2011; Li et al., 2011; Liu et al., 2011). In addition, ATF5 is overexpressed in several cancer cells, including glioma cells and breast cancer cells, and regulates cancer cell survival (Angelastro et al., 2006; Monaco et al., 2007; Li et al., 2009). ATF5 mRNA contains two upstream open reading frames (uORF) in the 5′-untranslated region (5′-UTR), and the structure of these is essential for the expression of ATF5 proteins under stress conditions, such as endoplasmic reticulum (ER) stress and oxidative stress (Watatani et al., 2008; Zhou et al., 2008; Hatano et al., 2013). Under stress conditions, eukaryotic initiation factor 2 α subunit (eIF2α) phosphorylation induces the translation of ATF5. Thus, ATF5 is a stress responsive transcription factor.

ATF5 is widely expressed in the brain (Angelastro et al., 2003; Wang et al., 2012; Torres-Peraza et al., 2013). In the embryonic brain, ATF5 is highly expressed in the ventricular zone (VZ) and the subventricular zone (SVZ) where neurogenesis occurs. In the adult brain, ATF5 is expressed in the cerebral cortex, striatum, hippocampus, cerebellum, and olfactory bulb (OB). In addition, ATF5 is expressed in neuronal progenitors and neurons, but not in glial cells. ATF5 is essential for proliferation and differentiation of progenitor cells and has a neuroprotective role against ER stress (Angelastro et al., 2003; Torres-Peraza et al., 2013). We and another group have recently identified the physiological roles of ATF5 in neuronal development using ATF5-deficient (ATF5^-/-^) mice (Wang et al., 2012; Umemura et al., 2015). Given that 70% of ATF5^-/-^ mice die within 3 days after birth, the neonatal lethality of ATF5^-/-^ mice was suppressed by hand-feeding of milk (Umemura et al., 2015).

Structural brain differences have been observed in ATF5^-/-^ mice compared with wild type littermates. The OB of ATF5^-/-^ mice was smaller than that of wild type littermates at both neonatal and adult stages, and had an irregular laminar structure (Umemura et al., 2015). The interneuron layer and olfactory nerve layer were also disrupted in the OB of ATF5^-/-^ mice. Both the number and ratio of proliferation cells of the OB and SVZ was reduced in ATF5^-/-^ mice (Umemura et al., 2015). Furthermore, in the olfactory sensory neuron of the MOE and the vomeronasal sensory neuron of the VNE in ATF5^-/-^ mice, neuronal maturation was impaired and increased apoptotic cell frequency was observed (Wang et al., 2012; Nakano et al., 2016).

ATF5^-/-^ male mice exhibited less aggressiveness when protecting their territory compared with wild type male mice. The aggression of male mice is evoked by pheromones in their urine (Chamero et al., 2007) and this pheromone is detected by the vomeronasal organ and MOE (Stowers et al., 2002; Mandiyan et al., 2005; Wang et al., 2006). Moreover, it has been reported that aggressive behavior can also be considered as a form of social communication (Koolhaas et al., 2013). ATF5 is most widely expressed in the brain and has several functions there. Thus, we speculate that ATF5 has an important role in higher brain regions, and ATF5-deficiency may contribute to abnormal behavior.

Monoamine neurotransmitters are involved in the modulation of cognitive processes including emotion, mood, memory, and learning. Dopamine (DA) is the main monoamine neurotransmitter and is involved in the modulation of anxiety-like behavior, memory, emotion, reward, and fear. Serotonin (5-HT) is involved in the modulation of cognition, anxiety, emotion, appetite, and sleep. Abnormalities of the monoamine neurotransmitter system contribute to the pathogenesis of several psychiatric disorders, including autism spectrum disorder (ASD), schizophrenia, depression, and anxiety disorder. Disturbances in monoamine neurotransmitter levels have also been observed in mouse models of mental disorders, and these appear to underlie several behavioral alterations.

In this study, to elucidate the influence of ATF5 deficiency on mouse behavior, we subjected ATF5^-/-^ mice to a comprehensive behavioral analysis. This analysis is useful for characterizing the involvement of specific genes and their function in the higher brain (Takao et al., 2007; Takao et al., 2010). We further explored the contribution of monoamine neurotransmitter levels in ATF5^-/-^ mice to behavioral outcomes. Our results demonstrated that ATF5^-/-^ mice exhibited abnormal locomotor activity in novel environments, abnormal anxiety-like behavior, reduced social interaction behavior, higher pain sensitivity, and reduced behavioral flexibility. We also showed that ATF5^-/-^ mice display perturbations in monoamine neurotransmitter levels in several brain regions. Our findings indicated that impairment of ATF5 function may be involved in the pathogenesis of psychiatric disorders.

## Materials and Methods

### Animals and Experimental Design for Comprehensive Behavioral Analysis

ATF5^-/-^ mice and their wild type (ATF5^+/+^) littermates were generated by mating heterozygous ATF5-deficient mice (ATF5^+/-^) (Umemura et al., 2015). Mice were genotyped using PCR after weaning (Umemura et al., 2015). These mice have been backcrossed into C57/BL6N for >19 generations. Neonatal death 3 days after birth was observed in 70% of ATF5^-/-^ mice (Umemura et al., 2015). Many ATF5^+/-^ mice pairs were mated, and surviving ATF5^-/-^ mice and their wild type littermates were used for behavioral analysis. The wild type littermates were randomly selected from the offspring.

The comprehensive behavioral analysis and study design have been described previously (Fujioka et al., 2014; Shoji et al., 2016). **Table 1** shows the behavioral tests conducted and the order in which they were performed. The behavioral test battery was performed with male mice aged 10–14 weeks old at the start of behavioral testing (*n* = 26 for both ATF5^-/-^ and ATF5^+/+^ mice). Each behavioral test was conducted on a separate day and all behavioral tests were performed once on each mouse. The order in which mice were subjected to behavioral tests was counterbalanced. For behavioral tests, mice were group-housed (two ATF5^+/+^ mice and two ATF5^-/-^ mice in a cage or one ATF5^+/+^ mouse and one ATF5^-/-^ mouse in a cage) in a room with 12 h of light and dark, with lights on at 7:00 a.m., and had access to food and water *ad libitum*. Before and after each mouse was tested, the apparatus for the behavioral tests was cleaned with diluted sodium hypochlorite solution to prevent a bias due to olfactory cues.

**Table 1:** Comprehensive behavioral analysis of ATF5^-/-^ mice.

Order	Test	Age ^∗^	Figures	Phenotype of ATF5^-/-^ mice
1	General health/neurological screen	10	**Figure 1**	Low weight
2	Light/dark transition	10	**Figure 2**	Hyperactive, abnormal anxiety-like behavior
3	Open field	10	**Figure 2**	abnormal locomotor activity, increased anxiety-like behavior
4	Elevated plus maze	11	**Figure 2**	Abnormal anxiety-like behavior
5	Hot plate	11	**Figure 6**	Pain sensitive
6	Social interaction in novel environment	11	**Figure 4**	NS
7	Rotarod	12	Data not shown	NS
8	Crawley’s social interaction test using three chambers	12	**Figure 3**	Abnormal social interaction, hyperactive
9	Prepulse inhibition/startle response	13	**Figure 8**	NS
10	Porsolt forced swim	13	**Figure 9**	NS
11	Gait analysis	14	Data not shown	NS
12	Eight-arm radial maze	18	**Figure 5**	NS
13	T-maze left-right discrimination test	24	**Figure 5**	Reduced behavioral flexibility
14	Tail suspension	29	**Figure 9**	NS
15	Cued and contextual fear conditioning	30	**Figure 6**	Hyperactive, pain sensitive
16	Social interaction in home cage	30	**Figure 4**	NS
17	24-h home cage monitoring	32	**Figure 7**	Disturbed circadian rhythm

*NS, no significant differences. ^∗^Age (weeks old) of the youngest mice at each test staring. The oldest mice are 1 month older than the age indicated.*

All behavioral tests were approved by the Animal Research Committee of the National Institute for Physiological Sciences. Animal experiments were approved by the Institutional Animal Experiment Committee of the university. Raw data from the behavioral tests, the date on which each experiment was performed, and the age of the mice at the time of the experiment are available in the Mouse Phenotype Database^1^.

### Neuromuscular Strength Test

Neuromuscular strength was assessed with the grip strength test and wire hang test as previously described (Shoji et al., 2016). A grip strength meter (O’Hara & Co., Tokyo, Japan) was used to estimate forelimb grip strength. Mice were lifted and held by their tail so that their forelimbs could grip a wire grid. The mice were then gently pulled backward by the tail until they released the wire grid. The peak force applied by the forelimbs of the mouse was recorded in Newtons (N). Each mouse was tested three times, and the highest value was used for statistical analysis. In the wire hang test, the mouse was placed on a wire mesh that was then inverted, and the latency to falling from the wire was recorded with a 60 s cutoff time.

### Light/Dark Transition Test

The light/dark transition test was performed as previously described (Takao and Miyakawa, 2006; Shoji et al., 2016). The apparatus used for the light/dark transition test consisted of a cage (21 cm × 42 cm × 25 cm) divided into two compartments of equal size by a partition with a door (O’Hara & Co., Tokyo, Japan). One compartment was brightly illuminated (380 lx), whereas the other compartment was dark (2 lx). Mice were placed in the dark compartment, and allowed to move freely between the two chambers with the door open for 10 min. The distance traveled, time spent in each compartment, total number of transitions between the light and dark chambers, and the latency of the first entrance to the light side were recorded automatically by ImageLD software (see Data analysis).

### Open Field Test

The open field test was performed as previously described (Tamada et al., 2010; Shoji et al., 2016). The apparatus used for the open field test consisted of an open box (40 cm × 40 cm × 30 cm). Mice were placed in the center of the apparatus and allowed to move freely. Data were collected for 120 min. Total distance traveled, vertical activity (rearing measured by counting the number of photobeam interruptions), time spent in the center area, and the beam-break counts for stereotypical behaviors were recorded automatically.

### Elevated Plus Maze Test

The elevated plus maze test was performed as previously described (Komada et al., 2008; Takao et al., 2016). The automated elevated plus maze apparatus consisted of two open arms (25 cm × 5 cm) and two closed arms of the same size, with 15 cm high transparent walls (O’Hara & Co.). Each mouse was placed in the center area of the maze (5 cm × 5 cm) facing one of the closed arms. The number of entries into each arm and the time spent in the open arm, closed arm, and center area was recorded for 10 min using ImageEP software (see Data analysis).

### Social Interaction Test in a Novel Environment

The social interaction test in a novel environment was performed as previously described (Shoji et al., 2016). Two same-genotype mice from distinct cages were placed into a box together (40 cm × 40 cm × 30 cm) and allowed to move freely for 10 min. The total duration of contacts, number of contacts, number of active contacts, and total distance traveled were recorded automatically using ImageSI software (see Data analysis). Active contact was defined as when two mice contacted each other and the distance traveled by each mouse was greater than 10 cm.

### Crawley’s Social Interaction Test Using Three Chambers (Sociability and Novel Preference)

The Crawley’s social interaction test using three chambers, a sociability and preference for social novelty test, was performed as previously described (Moy et al., 2004; Koshimizu et al., 2014; Ohashi et al., 2016). The apparatus comprised a three-chambered box (O’Hara & Co.). Each chamber was 20 cm × 40 cm × 22 cm and the dividing walls had small openings (5 cm × 3 cm) to allow exploration into each chamber. Data acquisition and analysis were performed automatically using ImageCSI (see Data analysis). The day before testing, the mice were individually placed in the middle chamber and allowed to freely explore the entire apparatus for 10 min.

In the sociability test, a stranger mouse (C57BL/6J male) that had no previous contact with the test mice was placed in a wire cage in one of the side chambers. The placement of the stranger mouse in the left or right side chambers was systematically alternated between trials. The test mouse was first placed in the middle chamber and allowed to explore the three chambers for 10 min. The amount of time spent in each chamber and time spent around each cage were recorded automatically for 10 min for the sociability test.

After the sociability test, each mouse performed the social novelty preference test for a further 10 min using a novel stranger mouse. The stranger mouse used for the sociability test stayed in the same wire cage (this mouse was considered as the familiar mouse) and a novel stranger mouse was placed in another cage on the opposite side of the chamber. The time spent in each chamber and the time spent around each cage were also recorded automatically for a second 10-min session for the social novelty preference test.

### Social Interaction Test in the Home Cage and 24-h Monitoring for Circadian Rhythm Analysis

Both a social interaction test in the home cage (Koshimizu et al., 2014; Ohashi et al., 2016) and 24-h monitoring for circadian rhythm analysis (Tamada et al., 2010) were performed as previously described.

To analyze social interactions and locomotor activity in the home cage, two same-genotype mice from distinct cages were placed into a cage together and monitored for 7 days. Social interaction behavior was measured by counting the number of particles. Two particles showed that the mice were not in contact and separate from each other. One particle showed that the two mice were in contact each other. Data acquisition and analysis were performed automatically using ImageHCSI (see Data analysis).

To analyze circadian rhythms, each mouse was individually placed in the home cage and distance traveled was measured automatically. The home cage was kept under 12-h light-dark cycle conditions (LD) for 8 days and subsequently kept under constant dark conditions (DD) for 11 days. Locomotor activity was recorded automatically. The circadian period was estimated for each mouse from the last 5 days of locomotor activity under DD.

### Eight-Arm Radial Maze Test

The eight-arm radial maze test was performed as previously described (Matsuo et al., 2010; Yasumura et al., 2014), using an automated eight-arm radial maze apparatus (O’Hara & Co.). Before pre-training, the mice were fed a controlled quantity of food to reduce body weight to 80–85% after gait analysis test for 10 days. This controlled feeding was continued throughout the test and the following T-maze test. After eight-arm radial maze test and T-maze test was completed, the next behavior test was performed after we stopped weight restriction and the mice returned to the original weight.

The mice were habituated to the eight-arm radial maze apparatus, and then pre-trained to consume a pellet from each food well. After pre-training, the test was performed. A test mouse was placed in the center area of the apparatus and allowed to move freely to consume all eight pellets within 25 min. One to two trials were performed per day (total 30 trials). During the 25–26th trial, a 30-s delay was introduced after four pellets had been consumed by confining the mice in the center area of the apparatus. During the 27–28th and 29–30th trials, the delay period was extended to 120 and 300 s, respectively. The number of different arm choices in the first eight entries, total number of revisits, time spent in the eight-arm radial maze apparatus, distance traveled, and number of omission errors were recorded. Data acquisition and analysis were performed automatically using ImageRM (see Data analysis). An arm visit was defined as traveling into the arm more than 5 cm from the central area of the apparatus.

### T-Maze Left–Right Discrimination Test

The T-maze left–right discrimination test was performed as previously described (Shoji et al., 2012; Yasumura et al., 2014). The mice were habituated to the T-maze apparatus and trained to consume a food pellet from the food pellet dispenser. For the test, the mice consumed the pellet on the right or left side of the T-maze (from the 1st to the 10th trial). From the 11th trial, the food pellet dispenser side was changed to the opposite side for reverse direction learning (from the 11th to the 20th trial). Correct responses, time in the T-maze apparatus, and distance traveled were also recorded. Data acquisition and analysis were performed automatically using ImageTM (see Data analysis).

### Contextual and Cued Fear Conditioning Test

The contextual and cued fear conditioning test was performed as previously described (Yasumura et al., 2014; Shoji et al., 2016). The conditioned stimulus (CS) was white noise played at 60 dB for 30 s. This was followed by a foot shock (0.5 mA, 2 s) as the unconditioned stimulus (US). Each mouse received three CS–US pairings with 2-min inter-stimulus intervals. Contextual testing was performed 24 h after conditioning. The freeze response of each mouse was recorded for 5 min in the same chamber. Cued testing with altered context was performed after contextual testing using a triangular box (35 cm × 35 cm × 40 cm) made of white opaque Plexiglas and located in a different room. Freezing behaviors were assessed during a 3-min free exploration, followed by a 3-min presentation of white noise. The percentage of time freezing in the conditioning phase, contextual phase, and cued phase was recorded. Distance traveled while receiving a foot shock was recorded in the conditioning phase. Data acquisition and analysis were performed automatically using ImageFZ (see Data analysis).

### Hot Plate Test

The hot plate test was performed as previously described (Takao et al., 2016). Mice were placed on a hot plate (Columbus Instruments, Columbus, OH, United States) preheated to 55.0 ± 0.3°C. Latency time to the first hind-paw response was recorded. The hind-paw response was defined as either a foot shake or a paw lick.

### Startle Response/Prepulse Inhibition Test

The startle response and prepulse inhibition test were performed as previously described (Fujioka et al., 2014; Shoji et al., 2016). A startle reflex measurement system (O’Hara & Co.) was used. Mice were placed in a Plexiglas cylinder and were left undisturbed for 10 min. The tests consisted of two test trials for the startle stimulus only and four test trails for prepulse inhibition. White noise (40 ms) was used as the startle stimulus for all trials. The startle response was recorded for 140 ms (measuring the response every 1 ms) starting with the onset of the prepulse stimulus. The background noise level in each chamber was 70 dB. The peak startle amplitude recorded during the 140 ms sampling window was used as the dependent variable. The intensity of the startle stimulus was 110 or 120 dB. The prepulse sound was presented 100 ms before the startle stimulus, and its intensity was 74 or 78 dB. Four combinations of prepulse and startle stimuli were employed (74–110, 78–110, 74–120, and 78–120 dB). The average inter-trial interval was 15 s (range 10–20 s).

### Porsolt Forced Swim Test

The Porsolt forced swim test was performed as previously described (Shoji et al., 2016). Four Plexiglas cylinders (20 cm height × 10 cm diameter) were used for the Porsolt forced swim test apparatus. The cylinders were filled with water (23°C) to a height of 7.5 cm. The mice were placed in the cylinders, and immobility and distance traveled were recorded for 10 min. Images were captured, and for each pair of successive frames, the area (pixels) within which the mouse moved was measured. When the area was below a certain threshold, mouse behavior was judged as “immobile.” Immobility lasting for less than 2 s was not included in the analysis. Data acquisition and analysis were performed automatically using ImagePS software (see Data analysis).

### Tail Suspension Test

The tail suspension test was performed as previously described (Onouchi et al., 2014; Shoji et al., 2016). The mice were suspended 30 cm above the floor in a visually isolated area by adhesive tape placed 1 cm from the tip of the tail, and their behavior was recorded for 10 min. Similar to the Porsolt forced swim test, immobility was judged by ImageTS software (see Data analysis) according to a certain threshold. Immobility lasting for less than 2 s was not included in the analysis.

### Measurement of Monoamine Neurotransmitters in Brain Tissue

To assess monoamine levels, mouse brains were harvested 8 days after all behavioral tests were completed. Adult mice (*n* = 8–15, 37–41 weeks old) were euthanized using cervical dislocation, and brains were rapidly frozen in powdered dry ice and stored at -80°C until slicing. The frozen brains were sliced at a thickness of 1 mm using a stainless steel slicer. The following 16 brain regions were microdissected using punching needles (0.5, 0.8, and 1 mm diameter) and kept at -80°C until sample preparation: medial prefrontal cortex (PFC), retrosplenial dysgranular cortex (RSD), nucleus accumbens (NAC), basolateral nuclei of the amygdala (BLA), dorsal hippocampus (Hip-D), ventral hippocampus (Hip-V), substantia nigra (SNR), ventral tegmental area (VTA), perirhinal cortex (PRh), median raphe nucleus (MnR), dorsal region of the dorsal raphe nucleus (DRD), sensory cortex (SC), motor cortex (MC), striatum (Stri), OB, and central part of the anterior hypothalamic area (AHC).

Tissues were homogenized in 0.1 M perchloric acid solution, including 2 mM sodium bisulfite and 0.02 mM EDTA, by sonication. The homogenates were centrifuged, and the precipitate was used for protein concentration determination and the supernatant was filtered through a 0.45 μm filter (Millipore, MA, United States). The filtrated samples were loaded into a high-performance liquid chromatography (HPLC) system (Waters, MA, United States), with the Acclaim 120 C18 Reversed-Phase LC Column (3.0 mm × 75 mm, Dionex, Sunnyvale, CA, United States) and the electrochemical detector (Model 5011 High sensitive analytical cell, Dionex). The mobile phase was composed of phospho-citric acid buffer (pH 3.0, 0.1 M), acetonitrile, and methanol [1000: 82: 200], with 0.012 M sodium 1-heptanesulfonate and 0.25 mM EDTA. Protein concentration was determined using the BCA Protein Assay kit (Thermo Scientific, Waltham, MA, United States).

### Data Analysis

Statistical analysis of behavioral test data was conducted using Stat View (SAS Institute, Cary, NC, United States). Data were analyzed using one-way or two-way ANOVA, two-way repeated ANOVA, Student’s *t*-test, or paired *t*-test where appropriate. All quantitative data are given as mean ± SEM. The significance level for statistical difference was set at *p* ≤ 0.05.

Behavioral data were obtained automatically by application software (ImageLD, EP, SI, CSI, HCSI, RM, TM, FZ, PS and TS) based on the public domain ImageJ program^2^, which was modified for each test by the authors. During the behavioral experiments, images of the mice were taken with a CCD camera and stored as sequential TIFF files. These files were analyzed automatically with software available from a public domain, NIH Image program (developed at the United States National Institutes of Health^3^). We also used ImageJ program^2^, which we modified for our purposes (Miyakawa and Crawley, 1999). Applications were specifically designed for each task as ImageLD, EP, SI, CSI, HCSI, RM, TM, FZ, PS, and TS. The correlation between the judgment of freezing by human observation and image analysis was greater than 0.95 during the fear conditioning test (Shoji et al., 2014), the Porsolt forced swim test, and the tail suspension test (data not shown). The correlation between values from the Accuscan system and the video tracking system for distance traveled was greater than 0.9 (Nakajima et al., 2008). These results support our use of the Image analysis software for behavioral phenotyping. ImageLD (Takao and Miyakawa, 2006), ImageEP (Komada et al., 2008), ImageTM (Shoji et al., 2012), and ImageFZ (Shoji et al., 2014) are freely available at the following URL: http://www.mouse-phenotype.org/software.html.

## Results

### General Characteristics of ATF5^-/-^ Mice

**FIGURE 1:**
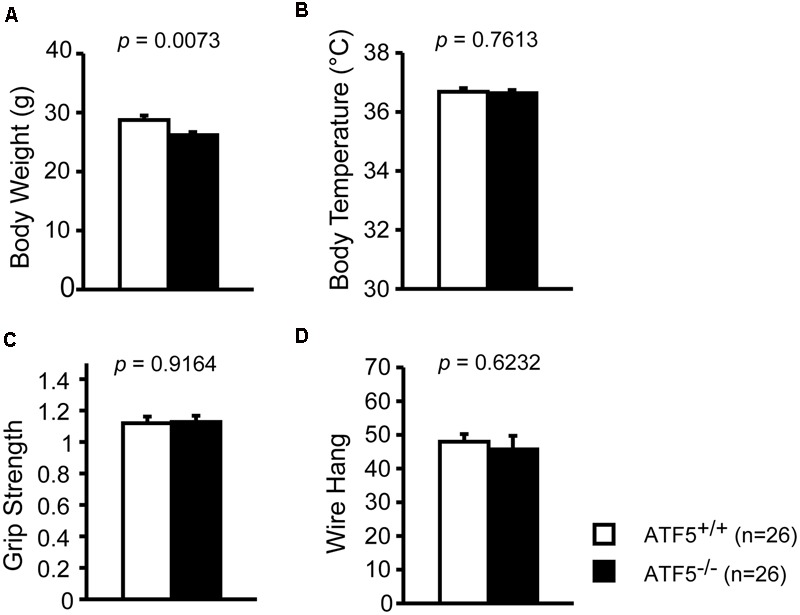
Physical characteristics of mice. We observed lower body weight but no abnormalities in physical characteristics of ATF5^-/-^ mice. Body weight **(A)**, body temperature **(B)**, grip strength test **(C)**, and wire hang test **(D)**. Data are given as mean ± SEM (*n* = 26).

We previously reported that 70% of ATF5^-/-^ mice exhibited neonatal death 3 days after birth (Umemura et al., 2015). To investigate the physiological role of ATF5^-/-^ mice in the higher brain, we performed comprehensive behavioral analyses using ATF5^-/-^ mice and wild type littermates (ATF5^-/-^ mice) (**Table 1**). There were no obvious differences in general health, including body temperature (**Figure 1B**), and coat and whisker condition between ATF5^+/+^ and ATF5^-/-^ mice, but body weight of ATF5^-/-^ mice was significantly lower than that of ATF5^+/+^ mice (**Figure 1A**). Muscle strength, which was measured by grip strength and wire hanging, was not significantly different between ATF5^+/+^ and ATF5^-/-^ mice (**Figures 1C,D**).

### Abnormal Locomotor Activity in Novel Environments and Abnormal Anxiety-Like Behavior of ATF5^-/-^ Mice

**FIGURE 2:**
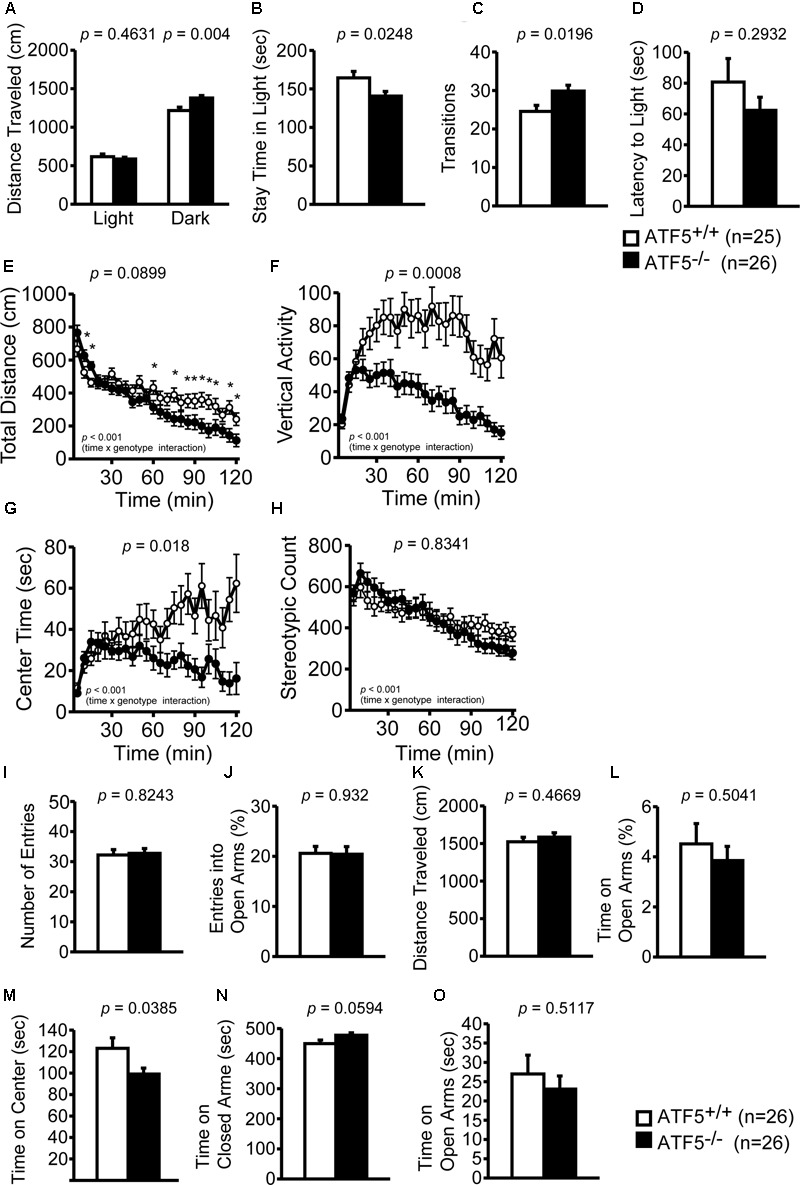
Abnormal locomotor activity and abnormal anxiety-like behavior was observed in ATF5^-/-^ mice. Light/dark transition test: distance traveled in the light and dark chamber **(A)**, time spent in the light chamber **(B)**, transition frequency between the light and dark chambers **(C)**, and latency to enter the light chamber **(D)**. The open field test: distance traveled **(E)**, vertical activity **(F)**, time spent in the center area **(G)**, and stereotypic behavior count **(H)**. There was a difference observed in the temporal changes in the four parameters during 120 min between ATF5^+/+^ and ATF5^-/-^ mice (time × genotype interaction, **E–H**). Elevated plus maze test: number of arm entries **(I)**, the percentage of entries into the open arms **(J)**, distance traveled **(K)**, percentage of time spent in the open arms **(L)**, time spent in the center area **(M)**, time spent in the closed arms **(N)**, and time spent in the open arms **(O)**. Data are given as mean ± SEM (*n* = 25 for ATF5^+/+^ mice and *n* = 26 for ATF5^-/-^ mice **(A–D)**; *n* = 26 for ATF5^+/+^ and ATF5^-/-^ mice **(E–O)**; ^∗^*p* < 0.05).

We examined locomotor activity and anxiety-like behavior of ATF5^-/-^ mice using the light/dark transition test, the open field test, and the elevated plus maze test. In the light/dark transition test, ATF5^-/-^ mice traveled for significantly longer distances in the dark compartment (**Figure 2A**) than did ATF5^+/+^ mice. ATF5^-/-^ mice also spent significantly less time in the light compartment (**Figure 2B**) and showed increased transition times (**Figure 2C**) compared with ATF5^+/+^ mice.

In the open field test, we examined the total distance traveled, vertical activity, time spent in the center area, and stereotypic counts for 120 min to investigate locomotor activity and anxiety-like behavior. The temporal changes of the four parameters during the 120 min demonstrated several differences between ATF5^+/+^ and ATF5^-/-^ mice (time × genotype interaction, **Figures 2E–H**). The total distance traveled (**Figure 2E**) and stereotypic counts (**Figure 2H**) during the first 15 min when the mice were exposed to a novel environment, were significantly higher in ATF5^-/-^ mice. The time spent in the center area was less in ATF5^-/-^ mice than in ATF5^+/+^ (**Figure 2G**). In particular, time spent in the center decreased significantly in the latter part of the 120-min test period. Similarly, the total distance (**Figure 2E**) and vertical activity (**Figure 2F**) of ATF5^-/-^ mice also decreased toward the end of testing. In the elevated plus maze, time spent in the center area of the elevated plus maze apparatus was lower (**Figure 2M**) and time spent in the open arm tended to be lower in ATF5^-/-^ mice than in ATF5^+/+^ mice (**Figure 2L**). These results suggested that ATF5^-/-^ mice display abnormal anxiety-like behavior and abnormal locomotor activity.

### Abnormal Social Interactions of ATF5^-/-^ Mice

**FIGURE 3:**
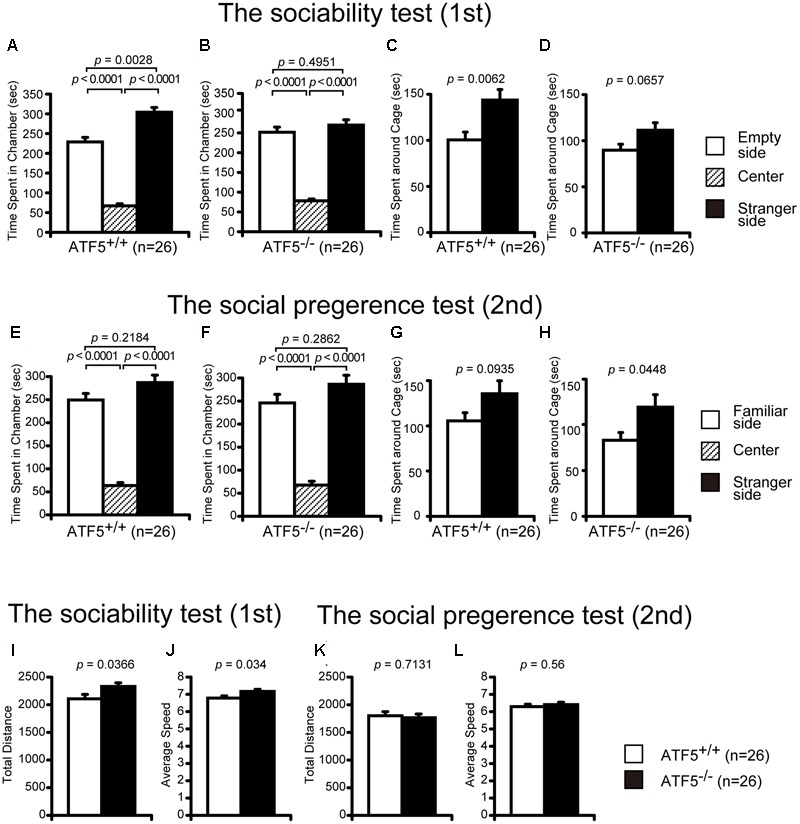
Impaired social interaction using Crawley’s social interaction test in ATF5^-/-^ mice. The sociability test was performed first **(A–D,I,J)**, followed by the novelty preference test **(E–H,K,L)**. Time spent in each chamber for ATF5^+/+^ mice **(A)** and ATF5^-/-^ mice **(B)**, time spent around each cage of ATF5^+/+^ mice **(C)** and ATF5^-/-^ mice **(D)** in the sociability test. Time spent in each chamber of ATF5^+/+^ mice **(E)** and ATF5^-/-^ mice **(F)**, time spent around each cage of ATF5^+/+^ mice **(G)** and ATF5^-/-^ mice **(H)** in the novelty preference test. Total distance **(I)** and average speed **(J)** in the sociability test, and total distance **(K)** and average speed **(L)** in the novelty preference test. Data are given as mean ± SEM (*n* = 13).

**FIGURE 4:**
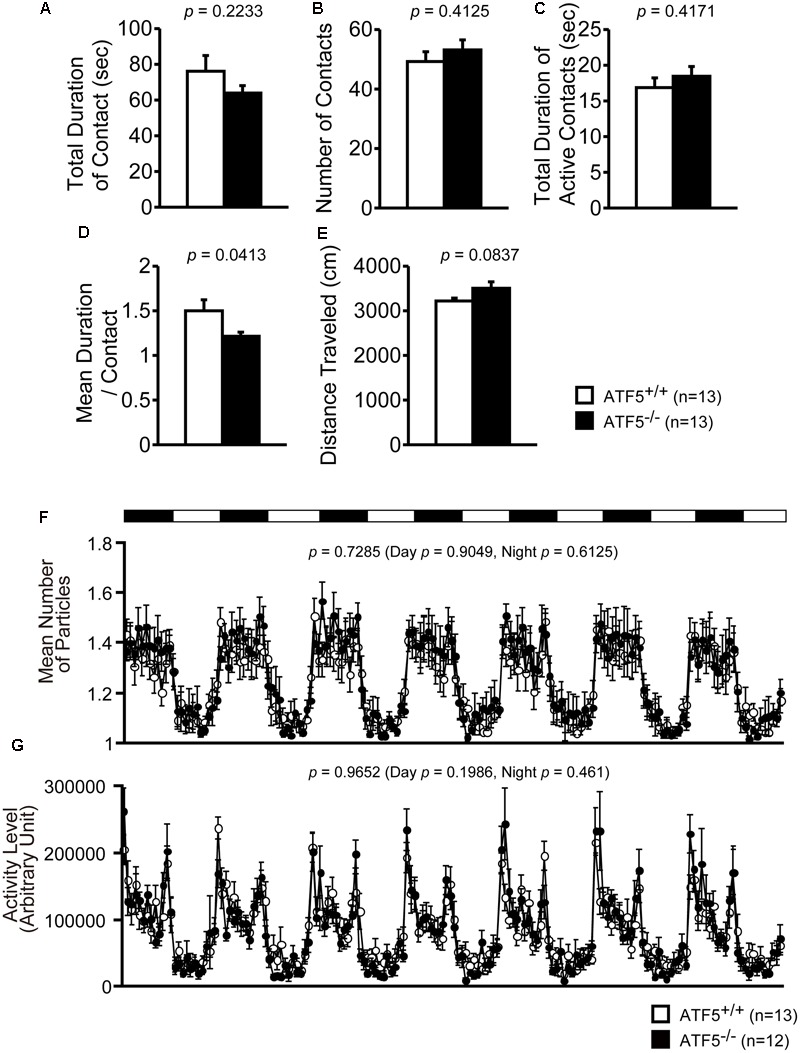
Normal social interaction in the home cage for 24-h monitoring in ATF5^-/-^ mice. Social interaction test in a novel environment: the interaction of two same-genotype mice from distinct cages was measured in a novel environment field for 10 min. The total duration of contact **(A)**, number of contacts **(B)**, the total duration of active contacts **(C)**, mean duration of one contact **(D)**, and distance traveled **(E)** are shown. Data are given as mean ± SEM (*n* = 13). Social interaction test in the home cage for 24-h monitoring: two same-genotype mice were placed into the same home cage and mouse activity was monitored 24 h a day for 1 week. The mice are displayed as particles, where one particle shows that two mice are in contact and two particles shows that two mice are separated in the home cage. Mean number of particles **(F)** and activity level **(G)** (*n* = 13 for ATF5^+/+^ mice and *n* = 12 for ATF5^-/-^ mice).

We examined the social behavior of ATF5^-/-^ mice using three tests: Crawley’s social interaction test using three chambers (**Figure 3**), the social interaction test in a novel environment (**Figures 4A–E**), and the social interaction test in the home cage (**Figures 4F,G**).

Crawley’s social interaction test using three cambers was used to assess sociability and social novelty preference. In the sociability test, a stranger mouse in a wire cage was placed in one side chamber and an empty wire cage was placed in the other side chamber. ATF5^+/+^ mice spent a significantly longer time in the chamber with the stranger cage compared with the chamber with the empty cage (**Figure 3A**). However, ATF5^-/-^ mice spent almost the same amount of time in the chamber between the stranger side and empty side (**Figure 3B**). Similarly, the time spent around the stranger mouse cage was comparable to the time spent around the empty cage in ATF5^-/-^ mice (**Figure 3D**), although time spent around the stranger mouse cage was longer than that of the empty cage in ATF5^+/+^ mice (**Figure 3C**). These results indicated that ATF5^+/+^ mice demonstrated a preference for the stranger mouse, which was not observed in ATF5^-/-^ mice, suggesting the latter exhibit decreased social interaction behavior. Moreover, ATF5^-/-^ mice exhibited a significantly longer total distance and faster average speed in the sociability test compared with ATF5^+/+^ mice (**Figures 3I,J**), suggesting that ATF5^-/-^ mice exhibit hyperactivity in novel environments.

After the sociability test, we performed a social novelty preference test. The stranger mouse used in the sociability test remained in the same wire cage, was now the familiar mouse (familiar side), while a novel mouse was placed in the other cage (stranger side). There was no significant difference in time spent in each chamber between ATF5^+/+^ and ATF5^-/-^ mice (**Figures 3E,F**). Time spent around the stranger mouse cage was a little longer than the time spent around the familiar mouse cage in ATF5^-/-^ mice, although there was no significant difference in time spent around the stranger mouse cage and familiar mouse cage in the ATF5^+/+^ mice (**Figures 3G,H**). These results suggest that ATF5^-/-^ mice exhibited similar novelty preferences to those in ATF5^+/+^ mice. There were no significant differences in total distance and average speed between ATF5^+/+^ and ATF5^-/-^ mice in the social novelty preference test (**Figures 3K,L**).

Next, we performed the social interaction test in a novel environment. Two same-genotype mice from distinct cages were placed in a novel environment and allow to interact with each other for 10 min. The mean duration of contact was significantly lower in ATF5^-/-^ mice (**Figure 4D**), although the total duration of contact, number of contacts, total duration of active contacts, and distance traveled were not significantly different from ATF5^+/+^ mice (**Figures 4A–C,E**). This suggests that ATF5^-/-^ mice exhibited hyperactivity in the novel environment.

Finally, we performed the social interaction test in the home cage for 1 week. Two same-genotype mice were placed in the same cage and their activity was recorded for a week. There were no significant differences in the values of mean number of particles or activity level between ATF5^+/+^ and ATF5^-/-^ mice (**Figures 4F,G**).

### Normal Spatial Working Memory, but Reduced Behavioral Flexibility in ATF5^-/-^ Mice

We performed the eight-arm radial maze test and T-maze right-left discrimination test to assess spatial working memory and reference memory of ATF5^-/-^ mice. Before pre-training for the eight-arm radial maze test, the mice were fed a controlled quantity of food pellets to reduce their body weight to 80–85%.

**FIGURE 5:**
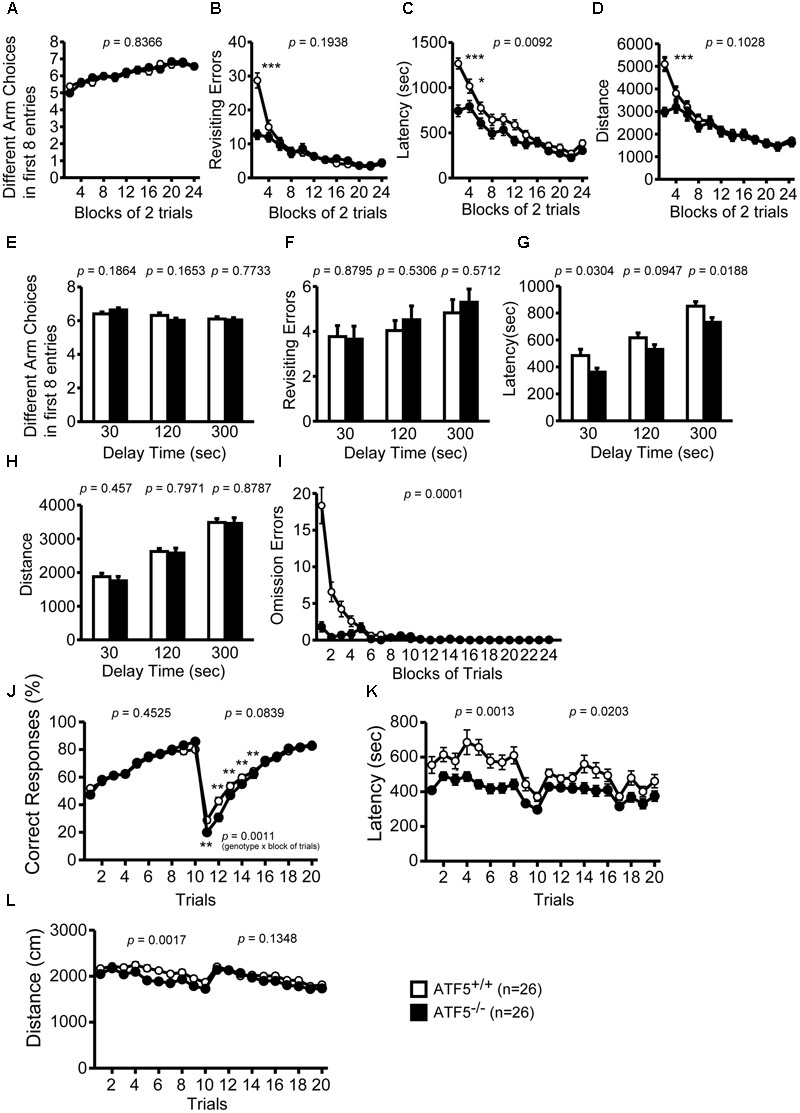
Abnormal behavioral flexibility, but normal spatial working memory in ATF5^-/-^ mice. Eight-arm radial maze test: the number of different arm choices in the first eight entries **(A,E)**, total number of revisits **(B,F)**, latency time in the eight-arm radial maze apparatus **(C,G)**, distance traveled **(D,H)**, and number of omission errors **(I)**. After training (trials 1–24), a delay was applied after the first four pellets were consumed **(E–H)**. T-maze left-right discrimination test: the percentage of correct responses **(J)**, the latency time in the T-maze apparatus **(K)**, and distance traveled **(L)**. In trials 1–10, the pellet dispenser side was pre-selected and in trials 11 to 20 this selection was reversed. Data are given as mean ± SEM (*n* = 26, ^∗^*p* < 0.05, ^∗∗^*p* < 0.01, ^∗∗∗^*p* < 0.001).

We observed no significant differences between ATF5^+/+^ and ATF5^-/-^ mice in the number of different arm choices in the first eight entries or the number of the revisiting errors, in which the mice returned to previously visited arms to retrieve a food pellet (**Figures 5A,B**). The latency and total distance to consume all eight pellets was lower in ATF5^-/-^ mice than in ATF5^+/+^ (**Figures 5C,D**), suggesting hyperactivity in ATF5^-/-^ mice. The number of revisiting errors, latency time to consume all eight pellets, and distance traveled were significantly lower in the first part of trials in ATF5^-/-^ mice compared with ATF5^+/+^ mice (trials 1–2 in **Figures 5B,D**, trials 1–4 in **Figure 5C**). These results were reflected in the lower number of omission errors in ATF5^-/-^ mice than in ATF5^+/+^ mice (**Figure 5I**).

We also performed the eight-arm radial maze test using longer delays, in which all the doors to arms were closed and opened at different times, and the mouse was confined to the center of the eight-arm radial maze apparatus for 30, 120, or 300 s after four food pellets were consumed. The number of different arm choices in the first eight entries and the number of revisiting errors with each delay were not significantly different between ATF5^+/+^ and ATF5^-/-^ mice (**Figures 5E–H**). These results suggest that ATF5 deficiency does not influence spatial working memory.

Next, we performed the T-maze left-right discrimination test to assess reference memory and behavior flexibility in ATF5^-/-^ mice. The ends of each side of the T-maze apparatus were equipped with an automatic pellet dispenser. All test mice were habituated to the apparatus and pre-trained to consume a food pellet from the pellet dispensers on the right or left side. In trials 1 to 10, the pellet dispenser was in the right or left side, and in trials 11 to 20, this selection was reversed. In the first 10 trials, there was no significant difference in the percentage of correct responses (choosing the correct side to consume the pellet) between ATF5^-/-^ mice and ATF5^+/+^ mice (**Figure 5J**), suggesting that reference memory in ATF5^-/-^ mice is comparable to that in ATF5^+/+^ mice. The location of pellet rewards was switched to the opposite side from trial 11. From trials 11 to 15, the percentage of correct responses in ATF5^-/-^ mice was significantly lower than that in ATF5^+/+^ mice, suggesting that ATF5^-/-^ mice had reduced behavioral flexibility. The latency to targets and distance traveled was lower in ATF5^-/-^ mice (**Figures 5K,L**). These results may reflect the hyperactivity of ATF5^-/-^ mice.

### Higher Sensitivity to Pain in ATF5^-/-^ Mice

**FIGURE 6:**
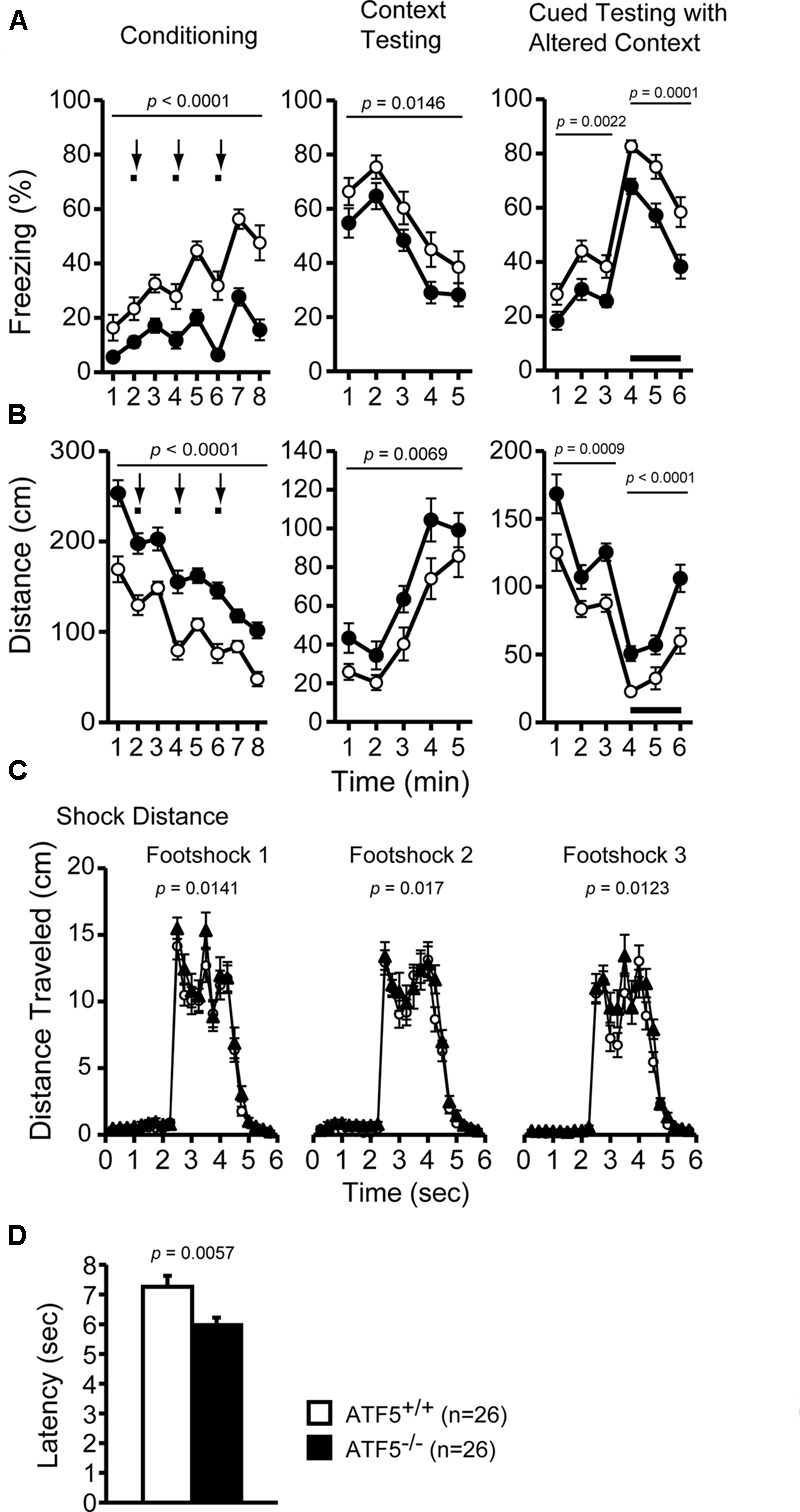
Higher pain stimulus sensitivity, but normal fear conditioning memory in ATF5^-/-^ mice. Cued and contextual fear conditioning test: the percentage of time freezing **(A)** and the distance traveled **(B)** in conditioning, context testing, and cued testing with altered context. Distance traveled during three foot shocks in the conditioning phase was recorded **(C)**. Bold lines and arrows represent tone and foot shock, respectively. The hot plate test: the latency to the first hind paw response on the hot plate at 55°C was measured to determine pain sensitivity in ATF5^-/-^ mice **(D)**. Data are given as mean ± SEM (*n* = 26, ^∗∗^*p* < 0.01, ^∗∗∗^*p* < 0.001).

We performed contextual and cued fear conditioning tests to assess fear memory in ATF5^-/-^ mice. The percentage of freezing was lower and the distance was greater in ATF5^-/-^ mice compared with ATF5^+/+^ mice in the conditioning, context test, and cued test. Although there were differences in amplitude of these variables, these patterns of change over time were similar in ATF5^+/+^ and ATF5^-/-^ mice, suggesting that fear conditioning in ATF5^-/-^ mice was not significantly different, but displayed hyperactivity. Distance traveled by ATF5^-/-^ mice during all three foot shocks was greater than that of ATF5^+/+^ mice. This result suggests that ATF5^-/-^ mice exhibit higher sensitivity to pain stimuli and hyperactivity (**Figures 6A,B**).

We performed the hot plate test to further assess pain stimulus sensitivity in ATF5^-/-^ mice. The latency to first hind paw response on the hot plate (preheated to 55°C) was shorter in ATF5^-/-^ mice than in ATF5^+/+^ mice (**Figure 6D**), suggesting that ATF5^-/-^ mice were more sensitive to pain stimuli.

### Abnormal Circadian Rhythms in ATF5^-/-^ Mice

**FIGURE 7:**
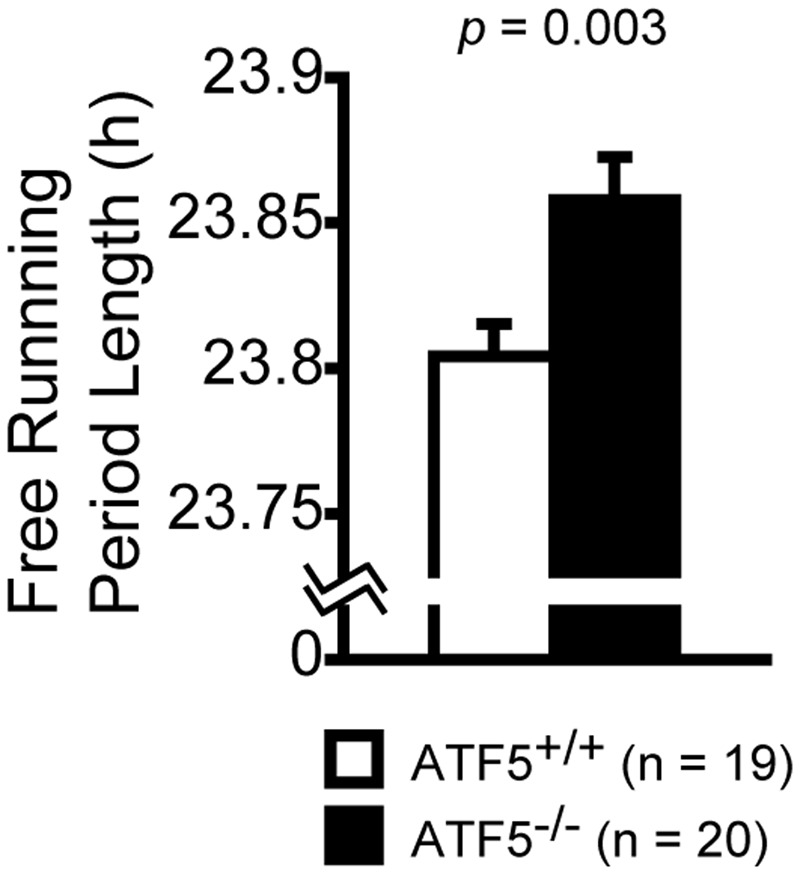
Disturbed circadian rhythms in ATF5^-/-^ mice. Each mouse was housed in the home cage and the locomotor activity was monitored for 24 h for circadian rhythm analysis. Initially, mice were trained for 8 days to a 12-h light/dark cycle (LD) and then kept in constant darkness conditions (DD) for the following 11 days. Free running period length was estimated from activity records under the DD conditions. Data are given as mean ± SEM (*n* = 19 for ATF5^+/+^ mice and *n* = 20 for ATF5^-/-^ mice).

We investigated circadian rhythms by monitoring locomotor activity in ATF5^-/-^ mice and ATF5^+/+^ mice. At first, the mice were habituated for 8 days to a 12-h light/dark cycle (LD). They were subsequently kept in constant dark conditions (DD) for the following 11 days. It has been reported that the internal circadian rhythm of mice is generally shorter than 24 h (Sadakata et al., 2007). Consequently, ATF5^+/+^ mice exhibited a shift to shorter sleep and wake rhythms under the DD condition. However, ATF5^-/-^ mice exhibited slightly, but significantly, longer circadian periods compared with ATF5^+/+^ mice (**Figure 7**).

### Normal Prepulse Inhibition and Depression-Like Behavior in ATF5^-/-^ Mice

**FIGURE 8:**
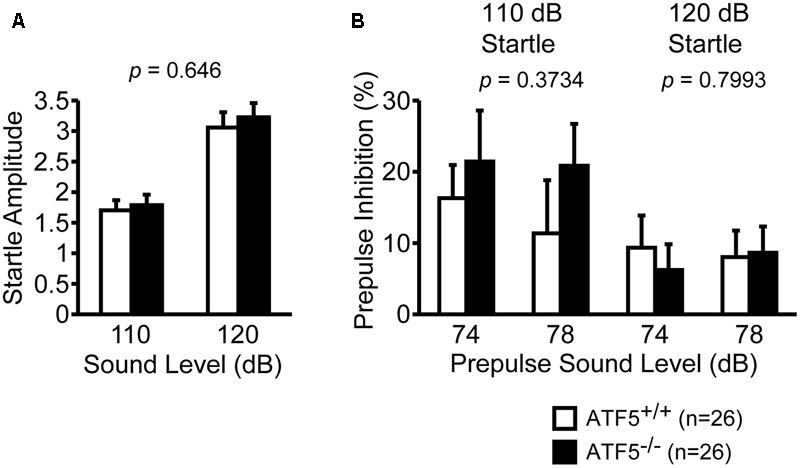
Normal startle response and prepulse inhibition in ATF5^-/-^ mice. The amplitude of startle response to the 110 and 120 dB acoustic stimuli **(A)**, and the percentage of prepulse inhibition of the 74 and 78 dB prepulse sound level **(B)**. Data are given as mean ± SEM (*n* = 26).

We performed the prepulse inhibition test to examine the efficiency of sensorimotor gating. There were no significant differences in the startle amplitude of the two sound levels (**Figure 8A**) or the percentage of prepulse inhibition of prepulse sound levels (**Figure 8B**) between ATF5^+/+^ and ATF5^-/-^ mice, suggesting normal sensorimotor gating in ATF5^-/-^ mice.

**FIGURE 9:**
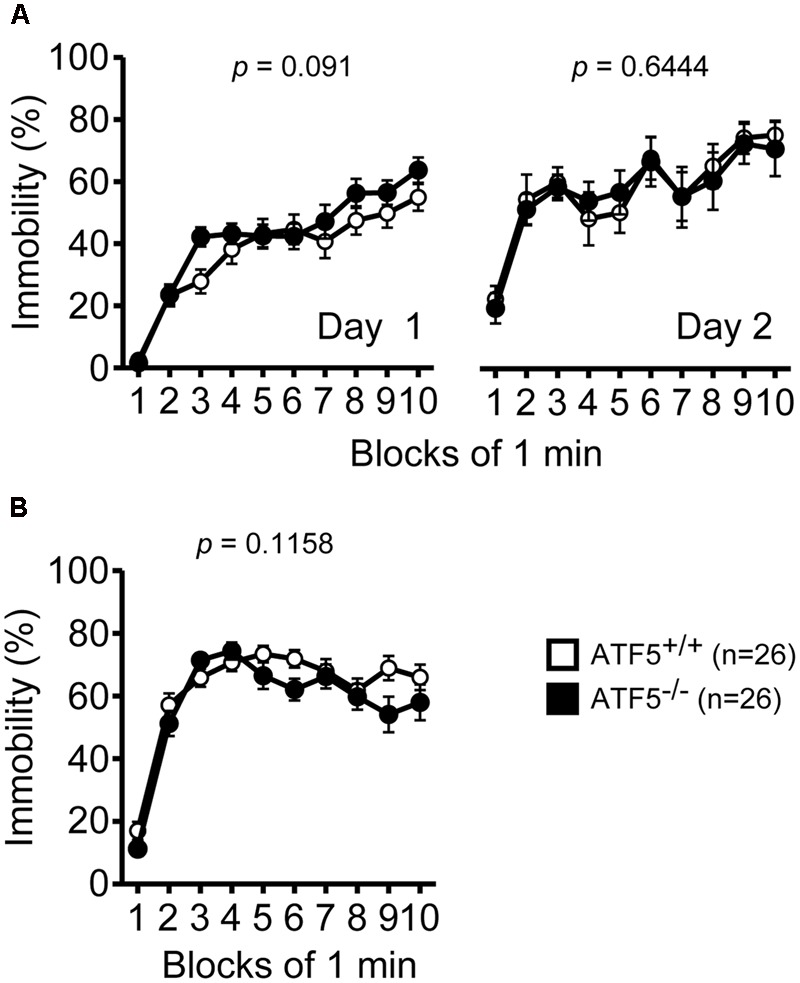
Normal depression-like behavior in ATF5^-/-^ mice. **(A)** The immobility time percentage was recorded for a 10-min period on days 1 and 2 in the Porsolt forced swim test. **(B)** The immobility time percentage was recorded for a 10-min period in the tail suspension test. Data are given as mean ± SEM (*n* = 26).

Next, we performed the Porsolt forced swim test and tail suspension test to assess depression-like behavior. There were no significant differences in immobility on day 1 or day 2 in the Porsolt forced swim test between ATF5^+/+^ and ATF5^-/-^ mice (**Figure 9A**). There was also no significant difference in immobility in the tail suspension test between ATF5^+/+^ and ATF5^-/-^ mice (**Figure 9B**). These results suggest that ATF5 deficiency does not elicit depression-like behavior.

### Altered Monoamine Neurotransmitter Levels in Multiple Brain Regions in ATF5^-/-^ Mice

The monoamine neurotransmitters 5-HT and DA affect rodent behavior, including social activity, anxiety-related behavior, and locomotor activity. To analyze monoamine levels in ATF5^-/-^ mice, we used HPLC to measure levels of monoamine neurotransmitters and their metabolites in 16 brain regions (listed above), after all the behavioral tests. ATF5^-/-^ mice demonstrated significant differences in levels of monoamine neurotransmitters and their metabolites in multiple brain regions compared with ATF5^+/+^ mice (**Table 2**). DA and its metabolites, 3,4-dihydroxy-phenylacetic acid (DOPAC) and homovanillic acid (HVA), were significantly lower in the BLA of ATF5^-/-^ mice than in ATF5^+/+^ mice. 5-HT and its metabolite, 5-hydroxyindole acetic acid (5-HIAA), were reduced in the DRD, although the apparent difference did not reach significance for 5-HT in this region (*p* = 0.075). The level of DA, HVA, and 5-HT, were perturbed in the OB of ATF5^-/-^ mice compared with ATF5^+/+^ mice. These results suggest that the level of monoamine neurotransmitters and their metabolites were disturbed in multiple brain regions of ATF5^-/-^ mice.

**Table 2:** Levels of monoamine neurotransmitters in individual brain regions.

Region	G	DOPAC	DA	HVA	5-HT	5-HIAA	3-MT
PFC	+/+	0.25 ± 0.03	0.52 ± 0.05	0.44 ± 0.05	8.58 ± 0.42	2.40 ± 0.22	nd
	-/-	0.24 ± 0.02	0.45 ± 0.07	0.43 ± 0.05	8.67 ± 0.58	2.50 ± 0.15	nd
RSD	+/+	0.12 ± 0.02	0.05 ± 0.03	0.19 ± 0.05	7.87 ± 0.85	2.40 ± 0.22	nd
	-/-	0.11 ± 0.02	0.03 ± 0.01	0.22 ± 0.05	6.74 ± 0.55	2.19 ± 0.16	nd
NAc	+/+	10.26 ± 0.87	101.89 ± 10.34	10.87 ± 1.01	10.01 ± 1.19	5.56 ± 0.63	5.82 ± 0.67
	-/-	11.55 ± 0.90	119.34 ± 15.86	11.07 ± 1.06	11.21 ± 0.76	5.84 ± 0.43	6.76 ± 0.54
BLA	+/+	4.23 ± 0.51	58.37 ± 15.86	6.91 ± 0.80	15.47 ± 2.47	5.84 ± 0.65	3.68 ± 0.73
	-/-	2.88 ± 0.27^∗^	23.60 ± 4.69^∗^	4.40 ± 0.42^∗∗^	19.01 ± 2.45	5.95 ± 0.49	2.06 ± 0.42
Hip-D	+/+	0.24 ± 0.04	0.70 ± 0.39	0.51 ± 0.06	15.16 ± 1.77	8.30 ± 0.83	nd
	-/-	0.26 ± 0.04	0.42 ± 0.18	0.49 ± 0.08	13.89 ± 0.85	8.04 ± 0.66	nd
Hip-V	+/+	0.30 ± 0.07	0.32 ± 0.14	0.33 ± 0.07	21.47 ± 1.18	9.04 ± 0.90	nd
	-/-	0.22 ± 0.03	0.23 ± 0.07	0.43 ± 0.09	23.39 ± 1.59	10.81 ± 0.85	nd
SNR	+/+	2.67 ± 0.29	8.45 ± 1.23	4.77 ± 0.45	32.44 ± 5.01	11.37 ± 0.87	0.86 ± 0.29
	-/-	2.37 ± 0.39	7.95 ± 1.62	4.59 ± 0.59	29.78 ± 4.61	10.90 ± 1.26	2.94 ± 2.02
VTA	+/+	5.65 ± 0.60	18.72 ± 2.27	6.50 ± 0.55	20.72 ± 2.51	12.13 ± 1.01	1.27 ± 0.36
	-/-	6.30 ± 0.64	20.14 ± 3.01	7.23 ± 0.66	22.54 ± 2.45	13.95 ± 1.62	1.56 ± 0.35
PRh	+/+	0.12 ± 0.05	0.70 ± 0.16	0.52 ± 0.16	13.36 ± 2.68	3.05 ± 0.53	nd
	-/-	0.10 ± 0.04	0.73 ± 0.13	0.36 ± 0.11	12.60 ± 1.72	3.04 ± 0.37	nd
MnR	+/+	1.96 ± 0.46	2.19 ± 1.03	4.11 ± 0.68	28.00 ± 3.50	24.06 ± 3.57	nd
	-/-	1.05 ± 0.16	0.63 ± 0.29	1.93 ± 0.34^∗^	26.60 ± 3.99	20.99 ± 2.44	nd
DRD	+/+	1.47 ± 0.16	2.57 ± 0.37	2.06 ± 0.40	48.37 ± 6.45	23.67 ± 2.56	nd
	-/-	1.38 ± 0.12	2.33 ± 0.29	1.43 ± 0.23	35.70 ± 3.46	17.39 ± 1.22^∗^	nd
SC	+/+	0.29 ± 0.03	nd	0.87 ± 0.10	7.35 ± 0.68	1.43 ± 0.10	nd
	-/-	0.29 ± 0.04	nd	0.92 ± 0.09	7.96 ± 0.69	1.46 ± 0.09	nd
MC	+/+	0.19 ± 0.02	0.07 ± 0.01	0.48 ± 0.05	10.38 ± 0.89	1.85 ± 0.18	nd
	-/-	0.19 ± 0.02	0.07 ± 0.02	0.46 ± 0.03	10.76 ± 0.89	1.90 ± 0.15	nd
Stri	+/+	15.84 ± 0.55	296.94 ± 21.99	21.47 ± 0.91	10.20 ± 1.22	6.00 ± 0.76	11.96 ± 0.60
	-/-	15.96 ± 1.09	282.74 ± 20.76	21.06 ± 1.44	8.11 ± 0.60	5.77 ± 0.36	10.91 ± 0.73
OB	+/+	1.24 ± 0.13	2.68 ± 0.14	1.92 ± 0.13	9.70 ± 1.09	2.81 ± 0.24	0.41 ± 0.06
	-/-	1.02 ± 0.08	3.46 ± 0.24^∗^	1.51 ± 0.10^∗^	14.46 ± 0.72^∗^	3.17 ± 0.12	0.45 ± 0.11
AHC	+/+	1.50 ± 0.12	4.82 ± 0.25	2.64 ± 0.33	26.60 ± 1.87	8.22 ± 0.41	0.53 ± 0.44
	-/-	1.45 ± 0.14	4.11 ± 0.59	2.01 ± 0.20	25.96 ± 2.61	8.39 ± 0.50	0.42 ± 0.30

*Levels of monoamines and their metabolites are expressed as pmol/mg protein. Values are mean ± SEM. ^∗^*p* < 0.05, ^∗∗^*p* < 0.01 vs. ATF5^+/+^ mice. +/+, ATF5^+/+^ mice; -/-, ATF5^-/-^ mice; G, genotype; DA, dopamine; DOPAC, dihydroxyphenylacetic acid; HVA, homovanillic acid; 5-HT, serotonin; 5-HIAA, 5-hydroxyindol acetic acid; 3-MT, 3-methoxytyramine; nd, not determined.*

## Discussion

ATF5 is widely expressed in the brain including the cerebral cortex, hippocampus, striatum, OB, and cerebellum (Angelastro et al., 2003; Wang et al., 2012; Dalton et al., 2013). In addition, ATF5 is important for proliferation and differentiation of progenitor cells, and has a neuroprotective role against ER stress (Angelastro et al., 2003; Torres-Peraza et al., 2013). In the present study, we performed a comprehensive behavioral analysis to explore the physiological functions of ATF5 in the higher brain. We previously reported that 70% of ATF5^-/-^ mice exhibited neonatal death 3 days after birth (Umemura et al., 2015). Many ATF5^+/-^ mice pairs were mated, and surviving ATF5^-/-^ mice and their wild type littermates were used for behavioral analysis. We performed a comprehensive behavioral analysis to investigate the physiological functions of ATF5 in the higher brain. This is useful to characterize the involvement of specific genes and their functions in the higher brain because the comprehensive behavioral analysis covers many distinct behavioral areas from simple sensorimotor functions to higher brain functions, including learning and memory. The behavioral tests were listed in order of level of stress induced and we conducted each test starting at the ages as shown in **Table 1**. After the stressful condition (i.e., food restriction or constant darkness condition for circadian rhythms analysis), we performed the following analysis after a certain period for recovery (above-mentioned). In this study, ATF5^-/-^ mice demonstrated behavioral abnormalities including hyperactivity in novel environments, abnormal anxiety-like behavior, reduced social interaction, higher pain sensitivity, disturbed circadian rhythms, and reduced behavioral flexibility. These results indicated that ATF5 is involved in the regulation of locomotor activity, anxiety, social interaction, circadian rhythm, and behavioral flexibility.

ATF5^-/-^ mice exhibited hyperactivity in a novel environment in the light/dark transition test (**Figures 2A,C**), the first 15 min of the open field test (**Figures 2E,H**), the sociability test of Crawley’s social interaction test (**Figures 3I,J**), the T-maze right-left discrimination test (**Figures 5K,L**), and the cued and contextual fear conditioning test (**Figure 6B**). The mice were exposed to novel environments in these five tests. In the social interaction test in a novel environment, the mean duration of contact was significantly lower in ATF5^-/-^ mice compared with ATF5^+/+^ mice (**Figure 4D**), indicating that they showed hyperactivity, rather than abnormal social interaction, in novel environments. In contrast, ATF5^-/-^ mice did not display hyperactivity in the social interaction test in the home cage (**Figure 4G**) or the open field test in the latter part of the 120-min test period (**Figures 2E,H**), where the test environments were familiar. These results indicate that ATF5^-/-^ mice may exhibit hyperactivity in novel environments. Moreover, ATF5^-/-^ mice demonstrated abnormal anxiety-like behavior in the light/dark transition test. The time spent in the light chamber was lower than for ATF5^+/+^ mice (**Figure 2B**), suggesting increased anxiety-like behavior. However, in ATF5^-/-^ mice, the number of transitions was greater (**Figure 2C**), which might suggest reduced anxiety-like behavior in ATF5^-/-^ mice or might reflect hyperactivity. ATF5^-/-^ mice spent less time in the center area in the open field test (**Figure 2G**) and in the center area in the elevated plus maze test (**Figure 2M**), suggesting increased anxiety-like behavior. Taken together, these results suggest that ATF5 deficiency enhances anxiety-like behavior.

We also observed that ATF5^-/-^ mice demonstrated abnormal social behavior (**Figures 3A–D**). Sociability assessed using Crawley’s social interaction test was lower in ATF5^-/-^ mice than in ATF5^+/+^ mice. It has previously been reported that Crawley’s social interaction test using three chambers is an appropriate approach to assess autistic-like behavior in mice (Moy et al., 2004). ASDs are neurodevelopmental disorders characterized by impairment of social interaction and verbal communication, ritualistic-repetitive behavior, and restricted interests (Levy et al., 2009; Lai et al., 2014). We demonstrated that the stereotypic count was also higher in ATF5^-/-^ mice than in ATF5^+/+^ mice during the first 15 min of the open field test (**Figure 2H**). Increased stereotypic activity in ATF5^-/-^ mice may be associated with stress-coping behavior, while ATF5^-/-^ mice also exhibited anxiety-like behavior. The ATF5^-/-^ mice exhibited lower behavioral flexibility than ATF5^+/+^ mice in the T-maze left-right discrimination test (**Figure 5J**). Moreover, it is reported that ASD patients frequently display disturbances in sleep and circadian rhythms (Glickman, 2010), and we observed disturbed circadian rhythms in ATF5^-/-^ mice (**Figure 7**). These observations provide evidence that ATF5 may be associated with the pathogenesis of autistic-like behavior.

Children with autism frequently present with different sensory symptoms not observed in children with typical development (Lane et al., 2010; Hazen et al., 2014; McCormick et al., 2016). Abnormalities in responses to sensory stimuli are highly prevalent in ASD and it has been reported that olfactory identification is impaired in children with autism (Bennetto et al., 2007; Kumazaki et al., 2016). Furthermore, atypical responsiveness to olfactory stimuli has been used to predict social impairment in children with ASD (Lane et al., 2010). In rodents, normal olfactory system development and olfactory function is important for social behavior (Silverman et al., 2010; Feierstein, 2012). It is reported that ATF5^-/-^ mice have olfactory system impairments (Wang et al., 2012; Umemura et al., 2015; Nakano et al., 2016). In ATF5^-/-^ mice, the size of the OB is reduced and the laminar structure of the OB is irregular (Umemura et al., 2015). Moreover, neuronal maturation is impaired in the MOE and VNE of ATF5^-/-^ mice (Wang et al., 2012; Nakano et al., 2016). ATF5^-/-^ male mice display reduced aggressiveness toward an intruder male mouse entering their territory. This male-male aggressive behavior is evoked by pheromones in the urine (Chamero et al., 2007), and therefore these differences may be due to olfactory impairments in ATF5^-/-^ mice. However, in the novelty preference test in Crawley’s social interaction test, there was no significant difference between ATF5^+/+^ and ATF5^-/-^ mice in novelty preference between the novel and familiar mouse (**Figures 3E–H**). These results indicate that there was no significant difference between ATF5^+/+^ and ATF5^-/-^ mice in olfactory function distinguishing between the novel and familiar mouse. Future work should investigate the ability of ATF5^-/-^ mice to detect odor and pheromones. In fact, olfactory abnormalities in children with ASD are still poorly understood compared with abnormalities of touch, vision, and hearing (Kumazaki et al., 2016). ATF5^-/-^ mice might be a useful tool to investigate the relationship between olfaction and social behavior in ASD.

Alterations of the serotonin system have also been reported in ASD (Anderson et al., 2009; Levy et al., 2009; Umeda et al., 2010; Lai et al., 2014). In addition, 5-HT levels were reduced in the brains and platelet-poor plasma of *patDp*/+ mice and *rSey^2^/+* rats, respectively, both of which are ASD models (Tamada et al., 2010; Umeda et al., 2010). We observed lower levels of 5-HT metabolites in the DRD of ATF5^-/-^ mice, suggesting they have serotoninergic system disturbances. We also demonstrated that ATF5^-/-^ mice exhibit perturbation of monoamine neurotransmitter levels in several brain regions. In particular, levels of dopamine and its metabolites in the BLA of ATF5^-/-^ mice were lower compared with those of ATF5^+/+^ mice (**Table 2**). Dopamine is involved in modulating locomotor activity, emotion, fear, reward, memory, and anxiety (Abraham et al., 2014). The dopaminergic system in the amygdala is important for modulation of anxiety-like behavior (Diaz et al., 2011). Mice exhibiting anxiety-like behavior indicate alterations of dopamine levels in the amygdala (Summavielle et al., 2002; Stein et al., 2006; Kataoka et al., 2011). The anxiety-like behavior of ATF5^-/-^ mice may therefore be due to changes in levels of dopamine and its metabolites in the BLA.

There was no significant difference in spatial working memory, spatial reference memory or fear memory between ATF5^+/+^ and ATF5^-/-^ mice, as shown by the results of the eight-arm radial maze test (**Figure 5**), the T-maze right-left discrimination test (**Figure 5**), and the contextual and cued fear conditioning test (**Figure 6**). Activating transcription factor 4 (ATF4) is a transcription factor of the CREB/ATF family and is highly homologous to ATF5. eIF2α phosphorylation induces the expression of the ATF4 protein under stress conditions, such as ER stress, hypoxia, and oxidative stress. It has been reported that ATF4 is a negative regulator of CREB-dependent memory consolidation (Silva et al., 1998; Pittenger et al., 2002; Jiang et al., 2010). ATF4-specific down-regulation in the hippocampus induces impairments of spatial memory and memory flexibility (Pasini et al., 2015). ATF5 is expressed in the hippocampal neurons (Torres-Peraza et al., 2013). ATF4 is known to play an important role in memory function, and therefore ATF5 may also be important for memory. Nonetheless, further research is needed to elucidate whether ATF5 affects memory consolidation and long-term memory.

In summary, ATF5^-/-^ mice exhibited behavioral abnormalities including hyperactivity in novel environments, increased anxiety-like behavior, reduced social interaction, higher pain sensitivity, and reduced behavior flexibility. ATF5^-/-^ mice may provide a useful model for the study of psychiatric disorder pathology, including ASD, anxiety disorder, hyperactivity disorder, and so on. Further study of ATF5^-/-^ mice may yield novel therapeutic strategies for the treatment of these psychiatric disorders.

## Author Contributions

MU, TO, and KT performed the behavior tests and analyzed the data under the supervision of TM and YT. MU and AM performed the monoamine measurements and analyzed the data. MU, HN, and YT developed the mice, materials, and analytical tools. MU and KT wrote the manuscript. YT designed the study. All authors discussed the results, and read and approved the final manuscript.

## Conflict of Interest Statement

The authors declare that the research was conducted in the absence of any commercial or financial relationships that could be construed as a potential conflict of interest.

## Footnotes

1 http://www.mouse-phenotype.org/

2 http://rsb.info.nih.gov/ij/

3 http://rsb.info.nih.gov/nih-image/
